# Personality disorders and therapist countertransference: A review

**DOI:** 10.1192/j.eurpsy.2021.1176

**Published:** 2021-08-13

**Authors:** I. Cuevas Iñiguez, M.D.C. Molina Lietor

**Affiliations:** Psiquiatría, Hospital Universitario Príncipe de Asturias, Alcalá de Henares, Spain

**Keywords:** personality disorder, Therapy, countertransference

## Abstract

**Introduction:**

The countertransference of the therapist is crucial in psychotherapy. Although the concept of countertransference arose from psychoanalysis, it is considered a construct that occurs in any type of therapy.

**Objectives:**

The purpose of this review is to examinate the relationship between personality disorders and countertransference.

**Methods:**

A systematic literature review was performed on PubMed using the keywords “personality disorder” and “countertransference”. All papers published between 2015 and 2020 were evaluated.

**Results:**

There were significant relationships between and patients’ personality disorders and patterns of countertransference. Criticized countertransference were associated with paranoid and antisocial personality disorders. Borderline personality disorders was related to helpless, overwhelmed, and overinvolved responses. Narcissistic and schizotypal personality disorders were associated with disengaged therapist responeses. Helpless countertransference was associated with schizoid personality disorders. Avoidant personality disorders was associated with postive responses. Special/overinvolved therapist responses were associated with obsessive-compulsive personality disorders

**Conclusions:**

Several studies suggest that therapists’ emotional responses occur consistently in any kind of therapy. Moreover, specific personality disorders are associated with concrete emotional responses. Therefore, therapists can make therapeutic and diagnostic use of their conuntertransference.

